# The Relationship between Habitual Breakfast Consumption Frequency and Academic Performance in British Adolescents

**DOI:** 10.3389/fpubh.2015.00068

**Published:** 2015-05-06

**Authors:** Katie Adolphus, Clare L. Lawton, Louise Dye

**Affiliations:** ^1^Human Appetite Research Unit, School of Psychology, University of Leeds, Leeds, UK

**Keywords:** breakfast, academic performance, adolescents, learning, cognitive abilities test

## Abstract

Breakfast has been shown to be beneficial for cognitive and academic performance in school children. However, there is a paucity of studies which examine the relationship between breakfast consumption and academic performance and a complete absence of studies in UK school children. The aim of this study, therefore, was to examine the association between habitual breakfast consumption frequency and Cognitive Abilities Test (CAT) performance, a reasoning test routinely used in UK schools. Adolescents aged 11–13 years (*n* = 292; males: 53.8%) completed a questionnaire to report usual weekly breakfast intake frequency. Breakfast was subjectively defined by the participants. Habitual weekly breakfast consumption frequency was categorized as rare (0–2 days), occasional (3–4 days), or frequent (5–7 days). Participants’ CAT performance was used as a proxy measure of academic performance. The CAT has three components: verbal, non-verbal, and quantitative reasoning. Normative standard age scores (SAS) for verbal, non-verbal, quantitative reasoning, and overall mean SAS were obtained from school records and hierarchical linear regression models were applied, adjusting for the confounders: gender, ethnicity, socio-economic status, English as an Additional Language, and body mass index. Habitual breakfast consumption frequency did not significantly predict any CAT SAS in all models (crude and adjusted). However, methodological considerations which could account for this disagreement with previous research, were identified. These included the isolation of school-day breakfast consumption, use of a standard definition of breakfast, and measurement of actual academic performance. The findings of the current study suggest more comprehensive ways in which future studies might investigate the relationship between habitual breakfast consumption and academic performance.

## Introduction

There has been widespread research interest in the possibility that breakfast can influence learning in children and adolescents. A good deal of research has considered the short-term (same morning) effects of breakfast on cognitive performance outcomes in controlled laboratory-based environments. In a systematic research review, Hoyland et al. (1) reviewed 45 studies examining the effects of breakfast on children’s and adolescents’ cognitive performance. Although this evidence was somewhat mixed, breakfast consumption appeared to have a positive acute effect on cognitive performance relative to breakfast omission in children. More recent evidence also supports the short-term beneficial effect of breakfast on cognitive function in children and adolescents (2–4). Therefore, breakfast consumption has the potential to affect cognitive processes in school children, which may benefit learning and academic performance. However, breakfast is frequently skipped by children and particularly adolescents aged ≥11 years (5).

Increasing breakfast consumption could be a useful public health, education enhancing intervention. However, far less research has considered the effects of breakfast on ecologically valid outcomes of academic performance compared with the relatively plentiful publications on cognitive performance. Therefore, assumptions about the benefits of breakfast for school children’s learning are based on evidence demonstrating acute effects of breakfast on school children’s cognitive test performance from laboratory-based studies. Our recent systematic review retrieved only 22 studies examining the effects of breakfast on children’s and adolescents’ academic performance (6). The habitual and acute effects of breakfast and the effects of school breakfast programs (SBPs) were considered. The academic performance outcomes employed by studies included either school grades or standardized achievement tests. Despite the paucity of studies, there was consistent evidence that habitual breakfast consumption (frequency and quality) and SBPs have a positive effect on children’s academic performance with clearest effects on mathematic and arithmetic grades in undernourished children. Increased frequency of habitual breakfast was consistently positively associated with academic performance. In addition, some evidence suggested that quality of habitual breakfast (food groups and energy) was positively related to academic performance (6).

The current study includes 11- to 13-year olds who are predominately from low socio-economic status (SES) backgrounds and of low academic ability. Two cross-sectional studies have demonstrated a consistent positive association between habitual breakfast consumption frequency and achievement test scores in children and adolescents of similar socio-demographic characteristics to the current study (7, 8). In the studies by Edwards et al. (7) and Acham et al. (8), associations were apparent in school children comparable to those in the current study, aged 9–15 years, from low SES backgrounds and/or of low academic ability. Acham et al. (8) demonstrated in a sample of 645 Ugandan 9- to 15-year olds who were mostly of low academic ability and low SES, that those who usually consumed breakfast and a mid-day meal were almost twice as likely to score highly on unstandardized achievement tests for English, mathematics, life skills, and oral comprehension compared to those who had only one meal. This association was specific to boys, and consuming breakfast alone was not associated with academic performance (8). A positive association between breakfast eating frequency and achievement test scores was also reported by Edwards et al. (7) in a sample of 800 American 11- to 13-year olds, of whom 20% were eligible for free or reduced price school meals, indicative of low SES. Higher mean mathematics Measure of Academic Progress (MAP) scores were associated with habitually eating breakfast (≥5 days/week) compared with less frequent consumption (<5 days/week) (7). No association was found between breakfast frequency and reading MAP scores. Moreover, a prospective cohort study failed to find a significant positive association between breakfast consumption frequency and scores on standardized achievement tests for reading, mathematics and science in 21,400 American 5- to 15-year olds (9). This study considered only breakfast that was eaten with the family rather than total breakfast intake. Although there are some discrepancies in the literature, habitual breakfast consumption could potentially impact upon meaningful and educationally significant outcomes.

Although studies on the association between breakfast consumption and academic performance have been conducted in children and adolescents across a range of ages, no study to date has examined this relationship in British school children or considered academic outcomes used in the British school system. Consequently, the current study aimed to extend previous work to include a sample of school pupils from a British school and to examine the association between breakfast consumption and Cognitive Abilities Test (CAT) performance, an assessment method routinely used in UK schools. The CAT is typically administered at the start of Year 7, when school children are aged 11–13 years, during the important transition point between primary and secondary education. In the current study, the CAT was considered a proxy measure of academic performance. CAT performance is strongly predictive of academic achievement (10, 11). The high correlation of CAT scores with subsequent achievement on school examinations including National Curriculum (NC) key stage tests and General Certificate of Secondary Education (GCSE) examinations (10, 11) suggests that CAT scores are an acceptable proxy of academic performance. One previous study has demonstrated an association between the quality of habitual breakfast consumption and performance on school administered reasoning tests using the Scholastic Aptitude Test (SAT). Habitually consuming a breakfast providing <20% of total energy needs was associated with poorer total SAT performance compared with higher energy breakfasts in 9- to 11-year olds (12). Based on the findings of our previous review (6), it was hypothesized that habitual breakfast skipping would be negatively associated with CAT scores in 11- to 13-year old adolescents, after adjustment for confounding variables.

## Materials and Methods

### Participants

The study sample consisted of males and females aged 11–13 years who were recruited to take part in the study from a British secondary school in Leeds. Ages 11–13 years correspond to compulsory secondary school Years 7 and 8 in the British school system, where Year 7 is the first year of secondary education. A total of 369 participants [males: 191 (51.8%); females: 178 (48.2%)] aged 12.08 ± 0.58 years were eligible to take part in this study. Of the 369 participants invited to take part, 77 (20.9%) returned incomplete questionnaires or did not complete any of the CAT subtests. These 77 participants were excluded. Hence, the final sample for analysis consisted of 292 participants. Of the 292 included participants, 15 returned incomplete data sets with respect to the CAT subtests and were therefore excluded from some, but not all, of the analyses.

### Design

The study conformed to an observational cross-sectional survey design. Cross-sectional survey data were collected through a self-report questionnaire on breakfast habits and from school records (demographic information and the CAT data) from 2010 to 2011.

### Measures

#### Socio-Demographic Measures

Demographic information on age, gender, ethnicity, free school meal (FSM) status and English as an additional language (EAL) status were gathered from school records. For ethnicity, the categories Asian and British Asian (18.8%), mixed ethnicity (5.1%), Black British/African/Caribbean (4.5%), and other ethnic background (3.1%) were collapsed due to infrequent occurrence. This provided a dichotomous ethnicity variable with participants coded as “White British” (68.5%) or “other ethnic background” (31.5%). FSM status was used as a proxy for SES. In England, pupils who are of compulsory school age in full time education are recorded as claiming FSMs if their parents/guardians receive certain support payments and have applied to their local education authority (LEA) to claim FSMs. Broadly, to be eligible for FSMs, pupils must be from families without a member working >24 h/week and/or from low or no income families with limited capital assets. FSM status is an acceptable proxy of SES and a valid indicator of low income families and is associated with parental education level (13, 14). Participants who were claiming FSMs were classified as low SES and participants who were not claiming FSMs were classified as middle-high SES. Approximately 68% of the study school’s pupils were claiming FSMs, a level considerably higher than the proportion of pupils claiming nationally and in the Leeds LEA in 2013 [16.0 and 19.4%, respectively; (15)]. The height and weight of each participant was measured by trained researchers to determine BMI SD scores (BMI SDS) and weight classification. BMI SDS were calculated using the LMS growth Microsoft Excel add-in which expresses BMI as an SDS based on British 1990 growth reference data (16, 17). The Department of Health’s epidemiological cut-offs were used to define overweight and obesity as the 85th and 95th centiles (*z* scores 1.04 and 1.64, respectively) on the UK 1990 BMI reference curves (17).

#### Habitual Breakfast Consumption

Participants completed a self-report written questionnaire which contained three items relating to the participants’ habitual breakfast consumption frequency and type. This study focused on the association between CAT performance and habitual breakfast consumption frequency (e.g., number of breakfast eating occasions per week). Participants’ habitual breakfast intake frequency (per week) was used to classify habitual breakfast consumption. Habitual breakfast intake frequency (per week) was assessed by the question: “How many times per week do you normally have breakfast?” with possible numerical responses: “0,” “1–2,” “3–4,” “5–6,” and “7.” Habitual breakfast consumption frequency was categorized as rare (0–2 days/week), occasional (3–4 days/week), or frequent (5–7 days/week).

#### Academic Performance

Participants’ CAT performance was used as a proxy measure of academic performance. CAT scores were obtained from school records. The CAT has six levels of difficulty coded A–F, standardized for school children aged 7 years 6 months to 15 years 9 months. Participants completed level D or E which, according to normative data, are suitable for school children aged 10 years 6 months to 12 years 11 months (school Year 7) and 11 years 6 months to 13 years 11 months (school Year 8), respectively (10). The CAT has three timed, multiple-choice test batteries which measure ability to reason with, and manipulate three types of symbols: symbols representing words, symbols representing quantities, and symbols representing spatial, geometric, or figural patterns. Each battery has three subtests that assess different aspects of that style of reasoning. These are aggregated to provide a standardized measure of verbal, non-verbal, and quantitative reasoning ability. A description of the complete CAT battery including abilities tested, time permitted, and scoring is provided in the Supplementary Material.

The CAT was administered by teachers in a formal group examination setting during the first school term in October 2010. Participants worked in silence, but questions were permitted. For all test sessions, no unexpected events or incidents were recorded. The CAT was completed in three timed sessions of approximately 30 min for each reasoning battery (see Supplementary Material). Standardized oral instructions were given at the beginning of each subtest. Each subtest began with an example question and practice questions to ensure that participants were familiar with the test layout and question format before they began the test. This also reduced test anxiety and procedural learning effects on initial questions within the subtests. Participants recorded their responses on optical mark recognition answer sheets which were scored by an external organization (GL Assessment, London).

Each subtest is standardized to a mean of 100 and SD of 15 based on normative population data from a representative sample of ≈16,000 British school children from 566 schools aged 7.6–15.9 years (10). A raw score was obtained for each CAT subtest. The three subtest scores were aggregated and converted into three normative standard age scores (SAS) for verbal, non-verbal, and quantitative reasoning. An overall mean SAS was also calculated as the average of the three standardized scores. SAS were calculated by comparing an adolescent’s raw score with the national standardization sample score adjusted for age and were calculated by an external organization (GL Assessment, London). The decision to use participants’ SAS rather than raw scores as outcomes was based on several factors. First, SAS allow for performance to be compared to the general population to place a pupil’s performance on a meaningful scale. Second, SAS are adjusted to take account of a pupil’s age at the time the test was taken. Third, SAS are comparable across CAT levels and therefore allowed the maximum number of cases to be included in the analysis. Finally, SAS are comparable across batteries to permit comparisons between the three domains assessed.

### Ethical considerations

Ethical approval was obtained from the School of Psychology Research Ethics Committee at the University of Leeds, UK. This study adopted a process of assent to determine whether potential participants and their parents/guardians were willing to take part in the study. A letter was sent home to the parents/guardians of the participating school pupils, containing a cover letter and information sheet for the parent/guardian and an information sheet for the adolescent participants who were all aged 11–13 years. These letters provided parents and potential participants with written information about the purpose of the study and requirements for participation. These documents also stated that parents/participants should contact the researchers, via email or telephone, with any questions or queries regarding the study. Parents/guardians were informed that if they were happy for their child to take part in the study they did not need to respond to the letter or notify the researchers, and consent (by a process of assent) was assumed. Alternatively, if parents/guardians were not happy for their child to participate in the study, they were requested to return a reply slip that was enclosed with the letter.

### Statistical analysis

Statistical analyses were performed using SPSS version 21 (SPSS, Inc., Chicago, IL, USA) and the significance level (α-level) was set as *p* < 0.05. Descriptive analyses of CAT performance are presented according to gender and are compared to the national standardization sample (10). All data were summarized and boxplots were produced to screen for outliers and check for normality of distribution. To assess differences in CAT performance in the current sample compared to the national standardization sample (10), one-sample *t*-tests were employed on SAS for verbal, non-verbal, quantitative, and overall mean SAS.

A series of hierarchical linear regression analyses were performed to examine whether habitual breakfast consumption was associated with CAT scores while controlling for the covariates. A series of potential confounders were included in the analyses which included: sex, ethnicity, SES, and EAL. Highly statistically significant sex differences in CAT scores have been reported in large samples (>500,000) of British 11- to 12-year-old school children (18, 19). There is consistent evidence that SES is a predictor of academic performance and cognitive ability (20–23). Due to the high proportion of participants with EAL, it was assumed that the sample had a wide range of language and reading abilities. It was, therefore, deemed appropriate to consider EAL as a confounding variable to reduce any additional variance arising from language ability, particularly on verbal subtests which are more vulnerable to such confounds. Having EAL can disproportionately influence performance on verbal reasoning subtests due to the demands placed on reading and familiarity with language (24). Ethnicity was also included as a covariate as evidence indicates large differences in attainment associated with ethnicity at age 11, 14, and 16 years (25–27). Preliminary regression analyses also indicated that BMI SDS significantly predicted CAT SAS, and was therefore included as a covariate in the analyses. All of these covariates are also related to breakfast consumption (5, 28–32). Four hierarchical multiple regression analyses were conducted for each CAT measure: verbal, non-verbal, quantitative, and overall mean SAS. The “frequent” habitual breakfast consumption category (5–7 days/week) was the reference category in all analyses. Variables were entered into the regression analyses in three blocks resulting in a series of models to explore the impact of confounders in the relationship between habitual breakfast consumption and CAT performance. Model 1 shows the crude coefficients [Unstandardised beta coefficients (B) and standardised beta coefficients (β)] for habitual breakfast consumption only. In model 2, adjustments were made for the socio-demographic covariates SES, ethnicity, sex, EAL, and BMI SDS resulting in adjusted coefficients (for B and β). In model 3, the variables included in model 2 were adjusted for and interaction terms were added to examine interactions between each socio-demographic variable and habitual breakfast consumption. For clarity, only habitual breakfast consumption categories and interaction terms are presented in the main results. The full regression models are shown in the Supplementary Material (Table S2 in Supplementary Material).

## Results

### Participant demographic characteristics

Participant demographic characteristics are shown in Table 1. The sample consisted of 292 participants [males: 157 (53.8%), females: 135 (46.2%)] aged 11–13 years. The sample was ethnically diverse, such that approximately two-thirds of the sample were White British [200 (68.5%)] with the remainder from other ethnic backgrounds. A relatively large proportion of the sample had EAL [79 (27.1%)]. A high proportion of the sample were classified as low SES [119 (40.8%)]. The BMI SDS varied widely with a mean BMI SDS of 0.80 ± 1.25. Three (1%) participants were classified as underweight. Most participants were classified as normal weight [183 (62.7%)], but a relatively large proportion of participants were either overweight [27 (9.2%)] or obese [79 (27.1%)].

**Table 1:** **Participant demographic characteristics**.

Demographic characteristics	*n* (%)
**Gender**
Male	157 (53.8)
Female	135 (46.2)
**Ethnicity**
White British	200 (68.5)
Other ethnic background	92 (31.5)
**School year group**
Year 7	155 (53.1)
Year 8	137 (46.9)
**SES**
Middle/high SES	173 (59.3)
Low SES	119 (40.8)
**EAL**
No	213 (73.0)
Yes	79 (27.1)
	**Mean (SD)**
**Age (years)**	12.05 (0.58)
**Height (cm)**	153.15 (8.64)
**Weight (kg)**	49.02 (13.42)
**BMI SDS**	0.80 (1.25)

### Habitual breakfast consumption

Participants were classified into three habitual breakfast consumption categories based on breakfast intake frequency per week. Participants’ habitual breakfast consumption is shown in Table 2. The majority of the participants were frequent breakfast consumers, consuming breakfast on most days of the week. However, approximately a third (31.5%) of participants rarely consumed breakfast (≤2 days/week).

**Table 2:** **Proportion of participants (*n*, %) who frequently, occasionally, or rarely consumed breakfast**.

Habitual breakfast consumption	*N*	%
Rare (0–2 days/week)	92	31.5
Occasional (3–4 days/week)	77	26.4
Frequent (5–7 days/weeks)	123	42.1

### Academic performance: CAT scores

**Figure 1:**
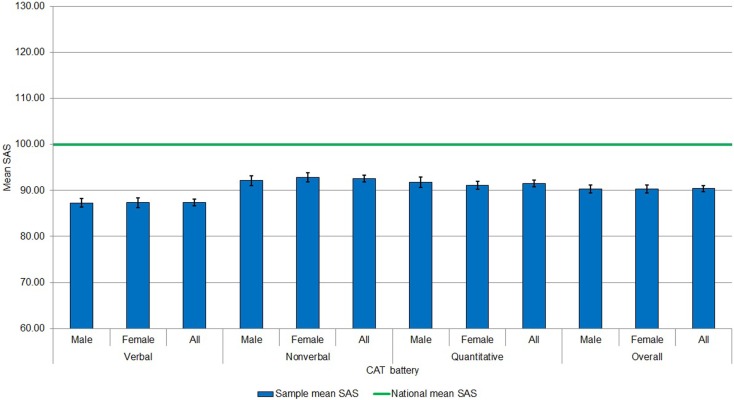
**Mean SAS by battery and overall for males, females, and all participants compared to the national mean SAS**.

Figure 1 shows mean SAS by battery and overall for males, females, and all participants compared to the national mean SAS (10). Mean verbal, non-verbal, quantitative, and overall SAS were significantly lower than the national mean, *t* (287) = −18.14; *t* (284) = −9.93; *t* (285) = −11.15; *t* (291) = −15.22; all *p* < 0.001, respectively). Comparing across domains, verbal reasoning ability was lower than non-verbal and quantitative reasoning ability, which may reflect the relatively high proportion of participants with EAL (27.1%) who may have lower English verbal ability. The current sample is not, therefore, representative of reasoning abilities among the general population and represents a low ability group, particularly for verbal reasoning.

### Associations between habitual breakfast consumption and CAT scores

Table 3 details the results of the hierarchical multiple regression for verbal, non-verbal, quantitative, and overall mean SAS. For verbal SAS, the crude model (model 1) was non-significant, *F*(2,286) = 1.08, *ns*. In model 2, the inclusion of socio-demographic covariates (Table 3; model 2) resulted in a significant model which explained 6.0% of the variance in verbal reasoning CAT SAS, *R*^2^ = 0.06; adjusted *R*^2^ = 0.03; *F*(7,281) = 2.36, *p* < 0.05. The change in variance (Δ*R*^2^) accounted for was 5.0% reflecting the effects of the addition of socio-demographic covariates, Δ*R*^2^ = 0.05; *F*(5,281) = 2.80, *p* < 0.05. Occasional and rare habitual breakfast consumption were not significantly associated with verbal reasoning SAS (Table 3; model 2). However, ethnicity and BMI were significant predictors of verbal SAS (full models shown in Table S2 in Supplementary Material). The standardized β suggests that verbal SAS were 0.17 SD lower in adolescents who were White British compared to those from any other ethnic background (β = −0.17, *p* < 0.01). Higher BMI SDS were predictive of better verbal SAS, such that verbal SAS increased by 0.13 SD with each SD increase in BMI SDS (β = −0.13, *p* < 0.05). In model 3, the inclusion of interaction terms (Table 3, model 3) did not significantly improve the model and all interaction terms were non-significant, *R*^2^ = 0.08; adjusted *R*^2^ = 0.02; *F*(17,271) = 1.26, *ns*. Correspondingly, the change in variance accounted for in model 3 was non-significant, Δ*R*^2^ = 0.01; *F*(10,271) = 0.51, *ns*. The relationship between rare and occasional habitual breakfast consumption and verbal reasoning CAT performance remained non-significant and this finding also did not vary by gender, ethnicity, SES, EAL status, or BMI SDS. Similarly, the significant relationship between ethnicity, BMI SDS and verbal SAS and the resulting adjusted β coefficients remained largely unaltered (β = −0.18, *p* < 0.01 and β = −0.13, *p* < 0.05, respectively; full models shown in Table S2 in Supplementary Material).

**Table 3:** **Hierarchical multiple regression analyses of the association between habitual breakfast consumption and CAT SAS**.

Model	Explanatory variables	Verbal CAT SAS			Non-verbal CAT SAS			Quantitative CAT SAS			Overall CAT SAS		
		B	SE B	β	B	SE B	β	B	SE B	β	B	SE B	β
1^a^	**Habitual school-day breakfast**												
	Frequent (reference)												
	Occasional	−2.57	1.79	−0.10	−1.76	1.96	−0.06	0.89	1.95	0.03	−1.22	1.64	−0.05
	Rare	−1.47	1.70	−0.06	−0.75	1.86	−0.03	−0.96	1.84	−0.04	−1.20	1.54	−0.05
2^b^	**Habitual school-day breakfast**												
	Frequent (reference)												
	Occasional	−1.72	1.78	−0.06	−1.85	1.96	−0.06	1.37	1.95	0.05	−0.77	1.64	−0.03
	Rare	−1.74	1.71	−0.07	−0.65	1.88	−0.02	−0.60	1.86	−0.02	−1.04	1.56	−0.05
3^c^	**Habitual school-day breakfast**												
	Frequent (reference)												
	Occasional	−1.50	1.85	−0.06	−2.20	2.03	−0.08	1.33	2.02	0.05	−0.83	1.70	−0.03
	Rare	−1.39	1.75	−0.05	−0.75	1.92	−0.03	−0.52	1.90	−0.02	−0.89	1.60	−0.04
	**Interaction terms**												
	Ethnicity × Occasional breakfast	1.39	3.87	0.03	6.02	4.28	0.10	3.81	4.23	0.07	3.47	3.55	0.07
	Ethnicity × Rare breakfast	−0.31	3.93	−0.01	−0.70	4.27	−0.01	−1.14	4.23	−0.02	−0.78	3.56	−0.02
	SES × Occasional breakfast	−0.49	3.87	−0.01	1.95	4.23	0.03	2.73	4.22	0.05	1.51	3.56	0.03
	SES × Rare breakfast	4.14	3.50	0.08	3.69	3.83	0.07	1.66	3.81	0.03	3.59	3.18	0.08
	Sex × Occasional breakfast	3.58	3.71	0.07	−3.04	4.07	−0.05	3.92	4.06	0.07	1.89	3.41	0.04
	Sex × Rare breakfast	0.08	3.48	0.00	−2.01	3.80	−0.04	0.15	3.79	0.00	−0.93	3.16	−0.02
	EAL × Occasional breakfast	−2.13	3.88	−0.04	−2.85	4.26	−0.05	−5.39	4.27	−0.09	−3.47	3.56	−0.07
	EAL × Rare breakfast	−3.72	3.99	−0.06	0.39	4.43	0.01	−2.67	4.38	−0.04	−1.80	3.66	−0.03
	BMI SDS × Occasional breakfast	1.06	1.50	0.05	1.68	1.65	0.07	1.73	1.63	0.07	1.45	1.38	0.07
	BMI SDS × Rare breakfast	−0.53	1.39	−0.03	1.60	1.54	0.07	0.80	1.52	0.04	0.49	1.27	0.03

*^a^Crude (unadjusted) model*.

^b^Adjusted model: includes habitual breakfast consumption adjusted for ethnicity, SES, sex, EAL, and BMI SDS

*^c^Fully adjusted model: includes habitual breakfast consumption adjusted for ethnicity, SES, sex, EAL, BMI SDS, and interaction terms*.

For non-verbal, quantitative, and overall CAT SAS, the same pattern of results was observed. The hierarchical multiple regression analysis for each outcome variable is shown in Table 3. Model 1 was non-significant, smallest *F*(2,284) = 0.40, *ns*. The addition of socio-demographic covariates in model 2 also resulted in a non-significant model, smallest *F*(7,279) = 1.61, *ns*. Model 3 was also non-significant, smallest *F*(17,269) = 0.97, *ns*. In all models (crude and adjusted), the resulting β coefficients indicated that habitual breakfast consumption did not predict non-verbal, quantitative, and overall reasoning CAT SAS.

## Discussion

### Overview of the findings

The study examined the association between habitual breakfast consumption frequency and CAT performance, a test of reasoning abilities taken by many school children in the UK in the first year of secondary education. Contrary to expectations, there was no evidence to support the hypothesis that habitual breakfast skipping is negatively associated with CAT performance in this sample of 11- to 13-year olds. The consistent null findings for verbal, non-verbal, quantitative, and overall reasoning ability indicates that frequency of habitual breakfast consumption did not influence performance on any CAT subtest.

The findings of the current study are inconsistent with the existing literature outlined in our previous systematic review which shows an overall positive association between breakfast consumption frequency and academic performance (6). Moreover, the findings are inconsistent with cross-sectional studies conducted in school children of similar socio-demographic backgrounds (7, 8). However, our findings concur with Miller et al. (9) whose study focused on breakfast that was eaten with the family rather than total breakfast intake. Furthermore, Miller et al. (9) employed a longitudinal design providing a stronger assessment of causality and controlled for a more extensive set of covariates in their analyses than most previous observational studies on breakfast and academic performance. For example, Edwards et al. (7) used stepwise regression analysis where theoretically important covariates such as SES, ethnicity, BMI, or other healthy lifestyle indicators are not always included. With this statistical approach, covariates are included in the final model on purely statistical grounds, resulting in a lack of control for confounders which may be linked but do not explain a statistically significant proportion of the variance in a particular sample. Given that many of the previous studies on breakfast and academic performance are cross-sectional, this suggests that the positive associations reported by some previous studies may be driven by residual or unmeasured confounding. Together with the lack of association found in the current study, this suggests that caution should be exercised when interpreting the positive associations between breakfast consumption and academic performance reported in previous work. Nevertheless, there are possible factors, which may explain the lack of significant associations found in the current study. These factors may also indicate important reasons for the discrepancy between the findings of the current study and other similar studies described above.

### Possible explanations for the null findings

#### The Proxy Measurement of Academic Performance

An important caveat to the results presented in this study is that academic performance was measured by reasoning tests that do not directly assess actual academic performance based on the content of the taught curriculum. Educational assessments in British secondary schools are either made by achievement tests, such as NC key stage tests and GCSE examinations, or by reasoning tests such as the CAT (11). The majority of previous studies that report positive associations between habitual breakfast consumption and academic performance use school grades or achievement tests that assess content drawn from the taught curriculum (6). Reasoning tests and achievement tests can be differentiated on a number of dimensions such as the content of the test and suitability for measuring academic progress overtime (11). This may account for the lack of association found in the current study and account for the disagreement with previous studies.

##### The predictive validity of reasoning tests for academic performance

Achievement tests are designed to measure specific outcomes of learning from the taught curriculum (11). All test content is directly drawn from what pupils learn at school and their outcomes reflect how well pupils have acquired and retained knowledge in key areas of the curriculum (11). These tests can therefore be considered as direct measures of academic performance. In contrast, reasoning tests contain more general assessments of content broadly similar to the taught curriculum using basic elements such as simple words or mathematical operators and shapes (11). Reasoning tests are considered to be valid predictors of academic performance. There is a strong correlation between CAT performance and subsequent attainment on NC key stage 3 tests (usually at age 14 years) and GCSE examinations [usually at age 16 years; (10, 11)]. Hence, in the present study, the CAT was considered as a good proxy measure of academic performance. However, while the correlations between CAT performance and NC key stage 3 and GCSE performance are all highly significant, this does not indicate a causal relationship. The indicated outcomes give a typical or most frequent outcome for a particular CAT SAS with some variation around this. Strand (11) demonstrated that approximately half of the variance in NC key stage 3 and GCSE outcomes is attributable to CAT performance at age 11 years. Clearly, other factors may influence performance on subsequent academic assessments. Such factors may include quality of teaching, opportunities to learn, parental support, motivation and effort of the pupil, and their emotional and physical well-being including nutritional intake. Hence, from the current study, it cannot be confidently concluded that habitual breakfast consumption has no association with academic performance given that a proxy indicator for direct measures of academic performance was used.

##### The consistency in reasoning test scores over time

Reasoning test scores tend to be more stable over time than achievement test scores. The CAT has good test–retest reliability based on data from over 10,000 UK school children who were tested at age 10 years (school Year 6) and 13 years [school Year 9; (24)]. The correlation coefficient for overall mean SAS at age 10 and 13 years was 0.89, suggesting a high degree of consistency in scores over time. However, despite high reliability coefficients, pupils’ scores between age 10 and 13 years can show significant progress over time [>10 standard score points; (24)]. In contrast, achievement tests, including NC tests and GCSE examinations, are used specifically to measure pupils’ progress over time (33). The consistency in reasoning scores over time suggests that school reasoning tests may not be sensitive to the potential beneficial effects of breakfast since scores tend to remain stable over time. Instead, achievement tests may be more sensitive measures in detecting an association between habitual breakfast consumption and academic performance as pupils’ performance generally progresses over time (33). Hence, achievement tests are likely to be influenced by the effects of habitually consuming breakfast to a greater degree than reasoning tests. The consistency in reasoning scores over time may account for the lack of associations reported in the current study, rather than the true absence of an association with academic performance.

Therefore, reasoning tests and achievement tests assess different domains (11). This suggests that the results of the present study may not permit conclusions about the association between habitual breakfast consumption and academic performance. By analyzing the association between habitual breakfast consumption and a proxy measure of academic performance, rather than measures of specific curricular attainment, this study may be understood as an extension of previous research, rather than a refutation of the previous positive associations reported [e.g., Ref. (7, 8)]. However, despite the issues surrounding the use of reasoning tests to measure academic performance, one previous study has demonstrated a positive association between breakfast consumption and performance on school reasoning tests (12). This suggests that factors other than the use of the CAT may account for the null findings reported in the current study. These possible factors are discussed below.

#### The Definition of Habitual Breakfast Consumption

One factor that may have affected the findings of the current study and contributed to the disparity between it and previous studies is the classification of habitual breakfast consumption. The literature gives mixed definitions and cut-offs to define “frequent” habitual breakfast consumption (34). In the present study, participants were classified into habitual breakfast consumption groups on a frequency basis where a specific number of days of breakfast intake per week were used to define rare, occasional, or frequent habitual breakfast consumption. Previous studies have also defined habitual breakfast consumption on a frequency basis. However, of the studies that do define habitual breakfast consumption on a frequency basis, there is variation in the frequency of breakfast intake to indicate the various consumption categories. For example, Lien (35) used a five-group classification system, which defined habitual breakfast consumption as never, 1–2 days/week, 3–4 days/week, 5–6 days/week, and every day. So (36) and Miller et al. (9) employed a seven-group classification system (0–7 days). Dichotomous classification systems are also employed to define habitual breakfast consumption as “regular” (≥5 days/week) or “irregular” [<5 days/week; (7, 37)]. One previous study employed a three category classification system to define habitual breakfast consumption comparable to the current study (38). However, Gajre et al. (38) used different frequencies of breakfast intake per week to indicate the various consumption categories (e.g., regular: ≥4 days/week, irregular: 2–3 days/week, and never: 0–1 day/week). While these are subtle differences in the frequency of breakfast intake, this may have affected the ability to detect a significant association with CAT performance in the present study.

#### The Lack of Distinction Between School-Day and Weekend Breakfast Intake

The present study did not distinguish between school-day and weekend-breakfast intake frequency in the classification of habitual breakfast consumption which may partly explain the non-significant associations with CAT performance. School-day breakfast intake has clear importance for academic performance given that it is consumed before school and so is likely to have immediate effects on the subsequent experience in school lessons. For example, where breakfast is consumed on one particular school morning, this may result in a short-term improvement in cognitive performance on the morning of consumption. Previous studies suggest that consuming breakfast has a modest short-term beneficial effect on cognitive function measured within 4 h post-ingestion in children and adolescents (2, 4, 39). If a child’s cognitive state improves, it is possible that they will begin to learn more during lessons which will accumulate over time to develop knowledge and skills they need in areas of the curriculum. Hence, it is plausible that the positive acute effects of school-day breakfast intake on cognitive performance translate, with repeated consumption, to cumulative effects on academic performance in the longer term. Furthermore, differentiating between school-day and weekend breakfast intake is important because habits may differ (40, 41). Weekend and school-day breakfast intake may also be different in terms of the time breakfast is consumed and the environment in which it is consumed because of different waking times and schedules. On weekends, more school children report consuming breakfast in general and more school children report eating breakfast with parents compared with school days (42). In the current study, the lack of distinction between school-day and weekend breakfast intake frequency may have resulted in a less relevant and sensitive measure of habitual breakfast consumption in relation to academic performance.

This lack of distinction will have also resulted in variation in the pattern of breakfast intake on school days and weekend days within each habitual breakfast consumption category. For example, a participant classified as a frequent breakfast consumer (≥5 days/week) could have consumed breakfast on three school days and two weekends or all five school days. An adolescent who habitually consumes breakfast on three school days is not indicative of frequent breakfast consumption on school days, which is most likely to influence academic performance. Moreover, adolescents within habitual breakfast consumption categories will not be entirely comparable in terms of their breakfast intake pattern. This variation in breakfast intake within the frequent, occasional, and rare consumption categories may account for the lack of associations found in the current study.

#### The Definition of a Breakfast Eating Occasion

Participants were not given a clear definition of breakfast meaning that breakfast was subjectively interpreted by the individual. What was considered as “breakfast” may have varied between participants in terms of the type and amount of food consumed, and the time of day. Some participants may have considered food consumed later in the morning, for example, at mid-morning break time, as breakfast, even though in these participants the overnight fasting period will have been extended for the majority of the morning lessons. Some participants may have also considered a very small amount of food or drink as breakfast. In addition, some participants may not have considered more unhealthy food items, non-traditional breakfast foods, food consumed on the way to school, or hand held food as breakfast.

The use of a questionnaire with a single item to measure habitual breakfast consumption as frequency per week did not allow for the assessment of the type and amount of food consumed, and the time of day it was consumed. Although participants were asked what they usually consumed at this time, this did not reflect daily differences in food intake at breakfast. Therefore, the data did not allow for the study to employ a standardized definition of breakfast *post hoc* (e.g., threshold amount of food or energy and/or time of day). This may have caused inconsistencies in habitual breakfast patterns between participants and contributed to the lack of significant association with CAT performance. Employing a dietary assessment method that permitted the measurement of food intake at breakfast would have allowed the composition and time of breakfast to be considered when defining a breakfast eating occasion. This would prevent very small breakfasts being classified as breakfast eating occasions and would differentiate breakfast from mid-morning snacks.

### Considerations for further work

The findings of the present study suggest that there are more comprehensive ways in which future studies might investigate the relationship between habitual breakfast consumption and academic performance. Future work should employ a measure of actual academic performance using achievement tests that assess outcomes of the taught curriculum. These measures may be more sensitive to the effects of habitual breakfast consumption. Assessing academic performance using measures of the taught curriculum would permit more confident conclusions about the relationship between habitual breakfast consumption and academic performance.

Further work should also employ more comprehensive dietary assessment methods to capture breakfast composition. A food diary or dietary recall method would allow for data on the composition of breakfast to be considered when classifying habitual breakfast consumption. These measures should include an adequate measurement period to reflect habitual breakfast consumption.

A breakfast eating occasion should be specifically defined to all participants to attempt to reduce inconsistencies between participants. This definition should also specify the time of day for the eating episode to be considered as breakfast. This will ensure that breakfast is not consumed late-morning thus resulting in an extended overnight fasting period. To strengthen this definition, future studies should apply a threshold indicator to define a breakfast eating occasion to prevent very small breakfast meals being classified as breakfast. The energy content of breakfast would be a useful objective indicator of a breakfast eating occasion.

School-day and weekend breakfast intake should be considered separately in the classification system used to define habitual breakfast consumption. This would provide a more appropriate measure of habitual breakfast consumption in relation to academic performance and account for differences in school-day and weekend breakfast intakes. By isolating school-day and weekend intake, there would be less variation within the categories representing frequent, occasional, or rare breakfast consumption. This would permit a more refined and relevant habitual breakfast consumption classification system.

## Conclusion

To conclude, the present study provided no evidence that habitual breakfast consumption was associated with a proxy measure of academic performance in the sample of 11- to 13-year-old adolescents studied. In drawing conclusions from this study, it is important to consider the proxy measure of academic performance utilized (i.e., the CAT). Although this study found no association between habitual breakfast consumption and CAT outcome variables, and differs from previous studies methodologically, it is premature to make firm conclusions about the value of habitual breakfast consumption for academic performance from this study. However, the present study has highlighted important methodological considerations that could be taken forward and applied to subsequent work in order to better understand the relationship between habitual breakfast consumption and academic performance.

## Author Contributions

KA conceived and designed the study, collected, analyzed and interpreted the data, and drafted and wrote the manuscript. LD and CL contributed to the design of the study, interpretation of the data, and revised the manuscript critically. All authors read and approved the final manuscript.

## Conflict of Interest Statement

Katie Adolphus declares that the research was conducted in the absence of any commercial or financial relationships that could be construed as a potential conflict of interest. Louise Dye and Clare L. Lawton have received funding from the food industry to examine the effects of food and food components including breakfast on cognitive function, satiety, glycemic response, and well-being but did not receive any support for this research.

## Supplementary Material

The Supplementary Material for this article can be found online at http://journal.frontiersin.org/article/10.3389/fpubh.2015.00068
