# Supplementary material for: The Relationship between Habitual Breakfast Consumption Frequency and Academic Performance in British Adolescents
Source: Front Public Health. 2015 May 6;3:68. doi: 10.3389/fpubh.2015.00068 (PMC4421928; doi:10.3389/fpubh.2015.00068)
Supplement: Supplementary file 1 [file Datasheet_1.PDF]

Supplementary Material

The relationship between habitual breakfast consumption frequency and academic performance in British adolescents

Katie Adolphus$_1$[1,*], Clare L. Lawton$_1$[1], Louise Dye$_1$[1]

[1]Human Appetite Research Unit, School of Psychology, University of Leeds, Leeds, LS2 9JT, UK

*** Correspondence:** Katie Adolphus, Human Appetite Research Unit, School of Psychology, University of Leeds, Leeds, LS2 9JT, UK
pskad@leeds.ac.uk

Keywords: Breakfast$_1$, academic performance$_2$, adolescents$_3$, learning$_4$, Cognitive Abilities Test$_5$.

1. Supplementary Figures and Tables

1.1. Supplementary Tables

Supplementary Table 1: Description of the complete CAT battery

CAT battery	Subtests	Description	Time (mins)	Number of questions	Max raw score
Verbal reasoning battery<br>Abilities tested:<br>• Ability to reason and manipulate symbols representing words	Verbal classification	Given three or four words belonging to one class, select which further word from a list of five belongs to the same class.	8	24	78
	Sentence completion	Select one word from a list of five to complete a sentence that is true and logical.	10	24	
	Verbal analogies	Determine the relationship between a pair of words. Decide which of five options would complete a second pair of words using the same relationship.	10	30	
Nonverbal reasoning battery<br>Abilities tested:<br>• Ability to reason and manipulate symbols representing spatial, geometric or figural patterns	Figure classification	Given three shapes belonging to one class, select which further shape from five choices belongs to the same class.	10	24	66
	Figure analogies	Determine the relationship between one pair of shapes. Decide which of five options would complete a second pair of shapes using the same relationship.	10	24	
	Figure analysis	Shown a figure of the method a square piece of paper was folded and where holes were punched through. Select which figure from five choices will resemble how the paper will look when it is unfolded.	10	18	
Quantitative reasoning battery<br>Abilities tested:<br>• Ability to reason	Number analogies	Determine the relationship between numbers in two example pairs. Select which of five options would complete a third pair of numbers using the same relationship.	12	20	58

CAT battery	Subtests	Description	Time (mins)	Number of questions	Max raw score
and manipulate symbols representing quantities	Number series	Determine the rule(s) for a number series. Select which number from a choice of five which completes the series using the same rule(s).	10	20	
	Equation building	Given 4-5 numbers and mathematical operators. Select one answer choice from five options that can be calculated by combining all the given elements to create a valid equation.	14	18	

Supplementary Table 2: Full hierarchical multiple regression models of the association between habitual breakfast consumption and CAT SAS

Model	Explanatory Variables	Verbal CAT SAS			Nonverbal CAT SAS			Quantitative CAT SAS			Overall CAT SAS		
		B	SE B	β	B	SE B	β	B	SE B	β	B	SE B	β
1[a]	**Habitual breakfast** (reference = Frequent)												
	Occasional	-2.57	1.79	-0.10	-1.76	1.96	-0.06	0.89	1.95	0.03	-1.22	1.64	-0.05
	Rare	-1.47	1.70	-0.06	-0.75	1.86	-0.03	-0.96	1.84	-0.04	-1.20	1.54	-0.05
2[b]	**Habitual breakfast** (reference = Frequent)												
	Occasional	-1.72	1.78	-0.06	-1.85	1.96	-0.06	1.37	1.95	0.05	-0.77	1.64	-0.03
	Rare	-1.74	1.71	-0.07	-0.65	1.88	-0.02	-0.60	1.86	-0.02	-1.04	1.56	-0.05
	**Ethnicity** (reference = White British)	-4.38	1.58	-0.17**	0.94	1.73	0.03	-2.29	1.71	-0.08	-2.10	1.44	-0.09
	**SES** (reference = Low)	1.99	1.48	0.08	2.90	1.62	0.11	3.52	1.62	0.14*	2.83	1.35	0.13*
	**Sex** (reference = Male)	0.98	1.46	0.04	1.58	1.60	0.06	0.02	1.59	0.00	0.79	1.33	0.04
	**EAL** (reference = No)	1.54	1.60	0.06	2.40	1.76	0.09	2.34	1.76	0.08	2.13	1.47	0.09
	**BMI SDS**	1.20	0.59	0.13*	1.32	0.65	0.13*	0.92	0.64	0.09	1.08	0.53	0.13*
3[c]	**Habitual breakfast** (reference = Frequent)												
	Occasional	-1.50	1.85	-0.06	-2.20	2.03	-0.08	1.33	2.02	0.05	-0.83	1.70	-0.03
	Rare	-1.39	1.75	-0.05	-0.75	1.92	-0.03	-0.52	1.90	-0.02	-0.89	1.60	-0.04
	**Ethnicity** (reference = White British)	-4.52	1.62	-0.18**	0.71	1.77	0.03	-2.67	1.76	-0.10	-2.41	1.48	-0.10
	**SES** (reference = Low)	1.96	1.52	0.08	2.51	1.66	0.10	3.53	1.65	0.14*	2.70	1.39	0.12*
	**Sex** (reference = Male)	0.85	1.48	0.04	1.35	1.63	0.05	-0.18	1.62	-0.01	0.65	1.36	0.03
	**EAL** (reference = No)	1.46	1.64	0.06	2.16	1.81	0.08	2.22	1.80	0.08	2.00	1.50	0.08
	**BMI SDS**	1.26	0.60	0.13*	1.26	0.66	0.12	0.88	0.65	0.09	1.07	0.55	0.12*
	Ethnicity * Occasional breakfast	1.39	3.87	0.03	6.02	4.28	0.10	3.81	4.23	0.07	3.47	3.55	0.07
	Ethnicity * Rare breakfast	-0.31	3.93	-0.01	-0.70	4.27	-0.01	-1.14	4.23	-0.02	-0.78	3.56	-0.02
	SES * Occasional breakfast	-0.49	3.87	-0.01	1.95	4.23	0.03	2.73	4.22	0.05	1.51	3.56	0.03
	SES * Rare breakfast	4.14	3.50	0.08	3.69	3.83	0.07	1.66	3.81	0.03	3.59	3.18	0.08
	Sex * Occasional breakfast	3.58	3.71	0.07	-3.04	4.07	-0.05	3.92	4.06	0.07	1.89	3.41	0.04
	Sex * Rare breakfast	0.08	3.48	0.00	-2.01	3.80	-0.04	0.15	3.79	0.00	-0.93	3.16	-0.02
	EAL* Occasional breakfast	-2.13	3.88	-0.04	-2.85	4.26	-0.05	-5.39	4.27	-0.09	-3.47	3.56	-0.07
	EAL * Rare breakfast	-3.72	3.99	-0.06	0.39	4.43	0.01	-2.67	4.38	-0.04	-1.80	3.66	-0.03
	BMI SDS* Occasional breakfast	1.06	1.50	0.05	1.68	1.65	0.07	1.73	1.63	0.07	1.45	1.38	0.07
	BMI SDS* Rare breakfast	-0.53	1.39	-0.03	1.60	1.54	0.07	0.80	1.52	0.04	0.49	1.27	0.03

[a] Crude (unadjusted) model; [b] Adjusted model; [c] Fully adjusted model; *p<0.05, ** p<0.01, ***p<0.001

2. References

Lohman, D. F., Thorndike, R. L., Hagen, E., Smith, P., Fernandes, C., & Strand, S. (2001). *Cognitive abilities test 3rd ed*. London: GL Assessment.
